# Pathogenic outcome following experimental infection of sheep with *Chlamydia abortus *variant strains LLG and POS

**DOI:** 10.1371/journal.pone.0177653

**Published:** 2017-05-11

**Authors:** Morag Livingstone, Nicholas Wheelhouse, Hannah Ensor, Mara Rocchi, Stephen Maley, Kevin Aitchison, Sean Wattegedera, Kim Wilson, Michelle Sait, Victoria Siarkou, Evangelia Vretou, Gary Entrican, Mark Dagleish, David Longbottom

**Affiliations:** 1 Moredun Research Institute, Penicuik, Midlothian, United Kingdom; 2 Biomathematics and Statistics Scotland, Edinburgh, United Kingdom; 3 Laboratory of Microbiology and Infectious Diseases, School of Veterinary Medicine, Faculty of Health Sciences, Aristotle University of Thessaloniki, Thessaloniki, Greece; 4 Formerly Laboratory of Biotechnology, Department of Microbiology, Hellenic Pasteur Institute, Athens, Greece; Veterinary Pathology, SWITZERLAND

## Abstract

This study investigated the pathogenesis of two variant strains (LLG and POS) of *Chlamydia abortus*, in comparison to a typical wild-type strain (S26/3) which is known to be responsible for late term abortion in small ruminants. Challenge with the three strains at mid-gestation resulted in similar pregnancy outcomes, with abortion occurring in approximately 50–60% of ewes with the mean gestational lengths also being similar. However, differences were observed in the severity of placental pathology, with infection appearing milder for strain LLG, which was reflected in the lower number of organisms shed in vaginal swabs post-partum and less gross pathology and organisms present in placental smears. Results for strain POS were somewhat different than LLG with a more focal restriction of infection observed. Post-abortion antibody responses revealed prominent differences in seropositivity to the major outer membrane protein (MOMP) present in elementary body (EB) preparations under denaturing conditions, most notably with anti-LLG and anti-POS convalescent sera where there was no or reduced detection of MOMP present in EBs derived from the three strains. These results and additional analysis of whole EB and chlamydial outer membrane complex preparations suggest that there are conformational differences in MOMP for the three strains. Overall, the results suggest that gross placental pathology and clinical outcome is not indicative of bacterial colonization and the severity of infection. The results also highlight potential conformational differences in MOMP epitopes that perhaps impact on disease diagnosis and the development of new vaccines.

## Introduction

Fetal mortality in sheep and goats caused by the obligate intracellular bacterium *Chlamydia abortus* (*C*. *abortus*) (syn. ovine enzootic abortion, OEA; enzootic abortion of ewes, EAE; ovine chlamydial abortion) was first described in 1950 [1] and is recognised as an economically important disease affecting the global agricultural industry. Characteristically, infection leads to abortion in the last 2 to 3 weeks of pregnancy or the birth of weak or stillborn lambs or kids. *C*. *abortus* also causes sporadic reproductive failure in cattle, horses and pigs and the bacterium presents a dangerous zoonotic risk to pregnant women [2–4].

The bacterium has a predilection for the trophoblast cells of the placenta where infection becomes established and then spreads out into the surrounding chorion. This leads to destruction of the placental tissue which affects nutrient acquisition and hormonal regulation and can result in the premature expulsion of the fetus [5]. Histological changes in the placenta and the appearance of lesions typically occur after 90 days gestation (dg) [6,7], however it has been shown that in experimentally-infected sheep, *C*. *abortus* can be detected in the placenta as early as 85dg [8].

*C*. *abortus* strains are considered to be homologous at the genomic and antigenic levels and have characteristic inclusion morphology [9]. However, two strains of *C*. *abortus*, LLG and POS, isolated from two geographically separate highland regions of Macedonia in Greece from an aborted goat fetus and aborted sheep fetus respectively [10], exhibit genetic heterogeneity [11–13]. These variant strains differ in their inclusion morphology, polypeptide profiles and reactivity to monoclonal antibodies against the major outer membrane protein (MOMP) when compared to typical ‘wild-type’ homologous *C*. *abortus* strains [9]. Interestingly, these strains are very different from other *C*. *abortus* strains circulating in Macedonia, or indeed other regions of Greece [9,10,11,14]

Studies in pregnant mice have shown these variant strains to be different from other *C*. *abortus* strains in terms of their ability to colonise the placenta. Reduced placental and fetal colonization is observed despite the presence of higher rates of abortion and reduced litter sizes [15]. Molecular rRNA secondary structure analysis, multilocus sequence typing (MLST) and multiple-locus variable-number tandem repeat (MLVA) analyses have demonstrated that the LLG and POS Greek variant strains are distinct and have their own *C*. *abortus* lineage [16,17]. Also recently, genome sequencing of LLG has revealed a number of differences in genes and proteins when compared to the wild type *C*. *abortus* reference strain S26/3 [14].

Despite the differences observed *in vitro* and in mouse *in vivo* studies, there are no reported studies on the pathogenesis of LLG or POS in small ruminants, or indeed whether LLG, which was isolated from a goat, also causes a similar disease in pregnant sheep. This study therefore aimed to investigate the pathogenesis of both Greek variant *C*. *abortus* strains in pregnant sheep in comparison to the typical wild type UK *C*. *abortus* reference strain S26/3.

## Materials and methods

### Ethics statement

This study was carried out in strict accordance with the Animals (Scientific Procedures) Act 1986 and in compliance with all UK Home Office Inspectorate regulations. The experimental protocol was approved by the Moredun Experiments and Ethical Review Committee (Permit number: E48/09). All animals were monitored throughout the study for any clinical signs at least three times daily and all findings recorded. Any animal found to be suffering or requiring treatment for example from secondary bacterial infections was given appropriate veterinary care (including use of antibiotics by a registered veterinary practitioner) in accordance with standard veterinary practice. All lambs born weak as a result of the challenge infection were independently assessed by a registered veterinary practitioner who took the decision to euthanize to end suffering based on the condition of the animal (criteria included, not able to stand or lift head but laying flat out on its side, not able or no interest in suckling, not opening eyes, laboured respiration, general minimal signs of life) by administration of an overdose of euthatal (sodium pentobarbital). All ewes and lambs were continually monitored and given appropriate veterinary care, where required, following parturition, at least three times a day until the end of the experiment, which was 2 months post lambing.

### Propagation of *C*. *abortus* strains

*C*. *abortus* strain S26/3, isolated from the placenta of a sheep that aborted as a result of EAE in Scotland [18] and strains LLG and POS, isolated in Greece from aborted goat and sheep fetuses respectively [10], were grown in fertile hens’ eggs and titrated in McCoy cells, as described previously [19]. Yolk sac inocula were stored in liquid nitrogen for subsequent infection of pregnant ewes. McCoy cells were used for the propagation of S26/3, LLG and POS for *in vitro* studies as follows: McCoy cells were grown in T_225_ flasks (Corning Costar, Scientific Laboratory Supplies Ltd, Nottingham, UK) and infected with *C*. *abortus* in fresh medium (RPMI 1640 medium + 2% fetal calf serum) containing 1μg/ml cycloheximide (Sigma-Aldrich Company ltd., Poole, UK), and grown at 37°C under 5% CO_2_ for 3 days. Infected cells were harvested using glass beads with vigorous shaking, and then centrifuged at 500 x *g* for 5 min to remove gross cellular debris. The supernatant was pelleted by centrifugation at 20,000 x *g* followed by purification of chlamydial elementary bodies (EBs) according to the method of Buendia et al. [20]. Protein concentrations were estimated using a Pierce™ BCA Protein Assay Kit (Fisher Scientific UK Ltd., Loughborough, UK).

### Experimental protocol

Forty Scottish Blackface sheep (aged 3–6 years) from an EAE-free flock and pre-screened for *C*. *abortus* antibodies by rOMP90-3 indirect ELISA [21] to ensure a *Chlamydia*-free status were randomly allocated to four groups, each containing 10 animals. Ewes were time-mated after synchronisation of oestrus with progesterone sponges (Veramix, Upjohn Ltd., Crawley, UK). Groups 1, 2 and 3 were challenged at day 70 of gestation with S26/3, LLG and POS, respectively. Inoculum was diluted with PBS to provide 2 x 10^6^ inclusion forming units (ifu) in each 2 mL dose and then administered by subcutaneous injection over the left prefemoral lymph node. Group 4 animals served as unchallenged negative controls. Each group was housed separately to avoid cross-infection and fed on a normal maintenance diet with free access to hay and water.

### Monitoring at lambing and abortion

The weight, sex, condition and time of delivery of each lamb or fetus was recorded. Abortion was defined as the production of dead lambs or of lambs dying within 48 hours of birth. Assistance in the survival of weakly lambs was limited to administration of the dam’s colostrum or of a colostrum substitute given by stomach tube or bottle, plus supplementary heat in the interests of animal welfare. Abortions were judged to be due to chlamydiosis if chlamydial EBs were demonstrable in the fetus, placenta or uterine discharges by stained smears, real time PCR or immunohistochemistry.

### Sample collection

Placentas were collected at lambing or abortion and examined for gross pathology typical of EAE and an assessment made as to percentage of placenta affected. Where gross pathology was evident, placental cotyledons were excised using disposable forceps and scalpel and placed in a sterile bijou for the subsequent preparation of modified Ziehl-Neelsen (mZN) smears. Where no gross placental pathology was observed, cotyledons were excised from three different areas of the placenta. Affected cotyledons plus surrounding intercotyledonary membrane were also collected and placed in 10% formol saline (FS) for histological examination and subsequent immunohistochemistry (IHC). Two vaginal swabs were collected from each animal at parturition for analysis by quantitative real-time polymerase chain reaction (qPCR). Blood samples were collected prior to challenge and at regular intervals thereafter during and following pregnancy for serological analysis.

### Assessment of gross placental pathology

Recovered placentas were inspected and assessed for the extent of gross pathology, as described previously [1,19,22,23], and to determine how typical they were compared to classical EAE presentation [5]. Placentas were oriented to ensure that the cotyledons were exposed and any obvious exudate cleared away to enable a thorough assessment of the extent of the lesions present on the cotyledons and intercotyledonary membranes. The whole surface of the placenta was examined and an estimate was made of the percentage coverage of the placental surface that was affected. Typically with this disease a very broad range of gross pathology can be observed with the same strain in different areas of the same placenta, as well as in multiple placentas from the same and different animals [1,22]. Such pathology, includes thickened and red intercotyledonary membranes and dark red cotyledons as well as cotyledons and intercotyledonary membranes that appear very necrotic and white in colouration. In addition some placentas can be covered or partially covered in a creamy viscous exudate that may appear off-white to yellow or pinkish in colour that adheres to the surface.

### Quantitative real-time PCR

The two vaginal swabs were vortexed vigorously in 1 mL phosphate buffered saline (PBS). All liquid was removed and centrifuged at 12,500 x *g* for 10 min in a standard bench-top microcentrifuge. DNA was extracted from the pellet using a DNeasy® Blood and Tissue Kit (Qiagen Ltd., Crawley, UK) according to manufacturer’s instructions and each sample eluted in 200 μL of elution buffer AE as supplied. Quantitative real-time PCR was carried out on DNA samples as described previously with minor modifications [24]. Briefly, the PCR reaction consisted of 12.5 μL of 2X TaqMan^®^ universal PCR master mix (Applied Biosystems, Warrington, UK), 300 nM final concentration of each primer (*OmpA* forward primer 5'-GCGGCATTCAACCTCGTT-3' and reverse primer, 5'-CCTTGAGTGATGCCTACATTGG-3'), 250 nM final concentration of fluorescent probe (TaqMan^®^ probe, 5'-TGTTAAAGGATCCTCCATAGCAGCTGATCAG-3') and 1μL swab DNA made up to a final volume of 25 μL with sterile deionised water. *C*. *abortus* S26/3 genomic DNA was extracted from purified elementary bodies using a DNeasy^®^ Blood & Tissue Kit and quantified using a NanoDrop ND-100 (NanoDrop Technologies, Wilmington, USA) for use as a quantitative standard. Amplification and detection were performed using an ABI Prism 7000 sequence detection system (Applied Biosystems), following manufacturer’s standard protocols. The thermal cycling conditions were 50°C for 2 min and 95°C for 10 min, followed by 45 cycles of 95°C for 15 s and 60°C for 1 min. Each sample was tested in triplicate.

### Serological analysis

Serum samples, prepared from blood collected throughout the study, were analysed by rOMP90B-3 indirect ELISA, as described previously [21]. Optical densities were normalized using positive and negative control sera and then expressed as a percentage of the positive control using the following formula:{(OD sample–OD negative control)/(OD positive control–OD negative control)} x 100.

### Immunoblotting

Approximately 100 μg purified *C*. *abortus* S26/3, LLG or POS EBs were subjected to SDS-polyacrylamide gel electrophoresis using 4–12% Invitrogen NuPAGE Bis-Tris polyacrylamide gels with NuPAGE MOPS SDS running buffer (ThermoFisher Scientific, UK) under reducing conditions after boiling in sample buffer containing 2% SDS and 5% 2-mercaptoethanol. Protein was transferred to a nitrocellulose membrane by semi-dry blotting and blocked with 5% non-fat dried milk (NFDM)/Tris-buffered saline (TBS), pH 7.6 at room temperature (RT) for 60 min. Blots were incubated with sheep sera diluted 1/100 in 5% NFDM/Tris-buffered saline/0.1% Tween 20 (TBST) for 60 min at RT. Bound antibody was detected using horse radish peroxidase-conjugated donkey anti-sheep IgG (Sigma-Aldrich Company Ltd., Poole, UK) diluted 1/1,000 in 5% NFDM/TBST at RT for 60 min, followed by 3,3′-diaminobenzidine tetrahydrochloride (Sigma ‘Fast’ DAB tablets) as the substrate.

Dot blots were carried out to evaluate reactivity to conformational epitopes by applying approximately 10 μg EBs directly onto a nitrocellulose membrane, allowed to air dry and following the procedure detailed above. Whole EBs were also subjected to treatment with 2% *N*-lauroylsarcosine and incubated at 37°C for 60 min to produce chlamydial outer membrane complex preparations (COMCs), in which the major outer membrane protein is known to retain conformational structure [25–27]. The COMC preparations were applied to a nitrocellulose membrane and probed with sheep sera as detailed above.

### Histopathological examination

Placental tissues were fixed in 10% FS for 4–10 days and then trimmed, processed routinely through graded alcohols and embedded in paraffin wax. Sections (4 μm) were mounted on glass microscope slides, stained with haematoxylin and eosin (HE) and examined by light microscopy for the presence of pathological changes. All slides were read blind.

### Detection of *C*. *abortus* by immunohistochemistry

Semi-serial sections of placental tissue samples stained with HE were deparaffinized in xylene and rehydrated through graded ethanols to 95%. After quenching of endogenous peroxidase activity with 3% hydrogen peroxide (Sigma) in methanol (v/v), the sections were washed in TBS, non-specific antibody binding blocked by incubation in 20% normal goat serum (NGS) in TBS for 30 min prior to overnight incubation at 4°C with a mouse monoclonal antibody (mAb) raised against the lipopolysaccharide (LPS) of *C*. *abortus* strain S26/3 (mAb 13/4, Santa Cruz Biotechnology, Heidelberg, Germany) diluted in 10% NGS/ TBS [2]. Visualisation of bound antibody was by the goat anti-mouse Envision™+ System HRP labeled polymer (Dako, Ely, UK) according to manufacturer’s instructions. The reaction was developed with the liquid DAB+ substrate system (Dako) for 5 min before counterstaining with haematoxylin, dehydration through graded alcohols, clearing and mounting. Negative control sections were prepared for each sample and treated identically except for substitution of the primary anti-chlamydial LPS 13/4 antibody with normal mouse IgG1 antibody (Dako). A positive control section, which comprised of placental tissue from a known ovine chlamydial abortion, was included in every run.

### Statistical analysis

Differences in the occurrence of abortion and of gross placental pathology between the three challenged groups were each investigated using permutation tests to obtain P values for Chi-squared statistics [28]. Quantitative PCR data (the mean of the triplicates) were log transformed (base exponential) and analysed using linear models with: ‘group’; presence or absence of abortion (‘abortion’); and presence or absence of gross pathology (‘gross pathology’) fitted as explanatory variables. Within the group variable contrasts of: control versus infection groups (S26/3, LLG and POS); LLG and POS groups combined versus S26/3 group; and LLG versus POS were fitted to answer specific questions of interest. Data was insufficient to include interactions and tests were based on coefficients estimated from the fitted model and thus are adjusted for all other terms in the model. Serology data were measured on ten occasions for each animal over the course of 22 weeks. These data were normalised using a log transformation (base exponential) and analysed using linear mixed models with sheep as a random effect and a power model specifying the covariance structure of the data. Models were built using data from week six of the study onwards to exclude the initial response to the challenge material. Fixed explanatory variables include ‘week’ to account for the temporal structure of the data, ‘group’ with the same contrasts as defined above, ‘abortion’, ‘gross pathology’ and appropriate two-way and three-way interactions. Approximate F tests were used to test the fixed effects in sequence in the order listed above and with the order of ‘abortion’ and ‘gross pathology’ reversed. All animals were treated as independent within groups. Chi-squared tests and analysis of qPCR data were performed using R version 3.3.1 and the serology analysis using GenStat 17^th^ Edition.

## Results

### Experimental infection and clinical outcomes

Subcutaneous challenge of ewes with all three *C*. *abortus* strains resulted in infection and abortion. The greatest number of abortions occurred in the S26/3-infected ewes (group 1 in Table 1), with the six ewes that aborted producing a total of nine dead fetuses and one non-viable lamb. In groups 2 (LLG) and 3 (POS), five ewes aborted in each resulting in six dead fetuses and one viable lamb apiece. One of the ten ewes in group 3 died from pneumonia prior to challenge and thus was removed from all analyses. Gestational ranges for all aborted ewes in the three challenge groups were similar, apart from one ewe in group 3 that aborted early at 108 days of gestation. Pearson’s Chi-squared permutation test revealed that there was no statistical significance in the number of abortions between groups 1, 2 and 3 (P = 1.000). There were no abortions in group 4 negative control animals which produced fifteen viable lambs. Ewes in groups 1 and 2 lambed over the typical gestational range, similar to those in group 4. However, ewes in group 3 lambed slightly earlier but within the normal gestational range (Table 1).

**Table 1:** The clinical outcome of pregnancy in ewes challenged with *C*. *abortus*.

	Number of ewes			Mean gestation in days (range)		Number of lambs		
Group	Pregnant	Lambed (%)	Aborted (%)	Lambed	Aborted	Viable	Non-viable	Dead
1	10	4 (40)	6 (60)	143 (142–145)	130 (120–139)	4	1^b^	9
2	10	5 (50)	5 (50)	146 (144–147)	131 (125–140)	8	0	6
3	10^a^	4 (44)	5 (56)	142 (139–144)	127 (108, 127–137)	6	0	6
4	10	10 (100)	0 (0)	147 (145–151)	n/a	15	0	0

Ewes challenged at 70 days gestation with *C*. *abortus* strains S26/3 (Group 1), LLG (Group 2) and POS (Group 3) at 70 days gestation or uninfected control ewes (Group 4).

^a^ One ewe died prior to challenge.

^b^ Neonatal death (born live but died within 48 hrs).

n/a, not applicable.

### Placental gross pathology and detection of *C*. *abortus*

Macroscopic lesions characteristic of EAE [1,5] were evident in placentas resulting from challenge with all three strains (Table 2). Overall, the placentas of the aborted and some of the lambed ewes showed gross abnormalities that differed in the degree, distribution and extent of coverage and this varied markedly from placenta to placenta of the same and different ewes (Table 2), as is typical of this disease [1]. However, the placentas obtained from some of the ewes that aborted and were infected with the two variant strains LLG and POS were more severely necrotic and were expelled after a longer time period following parturition than those infected with the wild-type strain S26/3, but the extent of the lesions appeared very similar to those observed with the wild-type strain.

**Table 2:** Gross pathology and detection of *C*. *abortus* organisms in placentas and vaginal swabs.

Group	Pregnancy Outcome	Ewe ID^a^	Extent of placental lesions (%)^b^		mZN^c^		qPCR^d^
			P1	P2	P1	P2	
1	Lambed	83D	0		-		2.4 x 10^3^
		210B	20		+		1.8 x 10^6^
		320B^e^	10		+		1.2 x 10^6^
		790A	0		-		7.1 x 10^2^
	Aborted	319B	100 [D]^f^	NF [D]^f^	+	NF	9.8 x 10^6^
		440B^e^	30 [D]^f^	80 [D]^f^	+	+	3.4 x 10^6^
		621B^e^	70 [D]^f^	NF [L]^f^	+	NF	2.7 x 10^7^
		715A	100 [D]^f^	100 [D]^f^	+	+	4.7 x 10^6^
		817A^e^	80		+		1.3 x 10^7^
		2604E	100		+		5.6 x 10^6^
2	Lambed	275B	0	NF	-	NF	3.8 x 10^2^
		605B	0		-		1.9 x 10^2^
		623B	0	0	-	-	2.7 x 10^1^
		992D	0		-		2.8 x 10^2^
		2646E	1		+		1.4 x 10^3^
	Aborted	336B^e^	100		+		5.2 x 10^6^
		449A^e^	10		+		1.2 x 10^6^
		451B	0 [L]^f^	10 [D]^f^	-	+	1.0 x 10^3^
		571A^e^	100N [D]^f^	100 [D]^f^	+	+	5.9 x 10^4^
		825A	100		+		4.5 x 10^3^
3	Lambed	374B	0		-		3.9 x 10^2^
		578B	15		+		8.9 x 10^2^
		744A^e^	30		+		4.5 x 10^5^
		2079E	5		+		2.6 x 10^6^
	Aborted	206B^e^	0 [L]^f^	100 [D]^f^	+	+	1.3 x 10^5^
		369B^e^	80		+		3.3 x 10^6^
		413B	100		+		2.2 x 10^5^
		748A	100		+		1.1 x 10^7^
		819A	40 [D]^f^	100 [D]^f^	+	+	5.1 x 10^6^

Gross pathology and detection of *C*. *abortus* organisms in placentas and genomic DNA in the vaginal swabs of ewes subcutaneously infected with *C*. *abortus* strains S26/3 (Group 1), LLG (Group 2) and POS (Group 3) at 70 days gestation. All Group 4 uninfected control ewes showed no evidence of any gross pathology and were all mZN and qPCR negative.

^a^ ewe identification numbers.

^b^ extent of gross placental pathology expressed as a percentage of the total surface area in placenta P1 (ewes with single lambs or first of twins) and P2 (ewes with second lambs).

^c^ detection of chlamydial EBs following mZN staining of impression smears from placentas P1 and P2: +, positive for chlamydial antigen; -, no EBs detected.

^d^ the number of *C*. *abortus* genomes detected per 1μl extracted total vaginal swab DNA following parturition for each ewe.

^e^ photographic examples of the placentas and/or fetuses are shown in S1 Fig (S26/3), S2 Fig (LLG) and S3 Fig (POS).

^f^ placentas from aborting ewes delivering twin lambs that were either dead [D] or living [L].

NF indicates were the placenta was not found for a particular ewe.

Some ewes produced twins where both aborted (eg 440B, 571A and 819A in Table 2) or produced one dead and one live lamb (e.g. 621B, 451B and 206B in Table 2). Aborted fetuses mostly appeared full term with a full coat (e.g. S3 Fig ewe 206B), while occasionally early death occurred in utero leading to expulsion of lambs devoid of hair and having a characteristic pot-bellied appearance (e.g. S2 Fig ewe 571A) [5]. The aborted fetuses in Group 2 from ewe 571A at 129 dg were devoid of hair and so likely died at a much earlier stage, which is supported by the autolytic nature of the placenta.

Macroscopic placental pathology, where evident, for all three groups consisted of necrosis of the cotyledons and inter-cotyledonary membranes, with affected cotyledons appearing darker red (e.g. S1 Fig ewe 440B and S3 Fig ewe 744A) or whitish (e.g. S1 Fig ewe 621B, S2 Fig ewe 336B and S3 Fig ewe 206B) in colouration, sometimes with flecks of exudate adherent. Inter-cotyledonary membranes consisted of partial and irregularly-shaped areas of thickening to total thickening of the whole placenta, and varied from oedematous (eg S1 Fig ewe 440B P2) to leather-like (e.g. S1 Fig ewe 621B, S2 Fig ewe 336B and S3 Fig ewe 369B) in appearance. As would be expected the extent and distribution of gross pathology varied between ewes that aborted versus those that lambed irrespective of the challenge strain, with coverage ranging from 0–30% for ewes that lambed versus 30–100% for ewes that aborted (Table 2). However, for some of the ewes challenged with strain LLG, gross pathology was much less evident. In particular, the lambed group appeared overall to be much less affected, with only a single ewe’s placenta (ewe 2646E in Table 2) showing any evidence of gross pathology and only a very small focal area (1%) affected, while two of the ewes in the aborted group had placentas exhibiting only 10% gross pathology (ewes 451B and 449A in Table 2). Across all three groups we observed an off-white to reddish dirty-coloured discharge adherent to the surface of some of the placentas, regardless of whether they came from aborted or lambed ewes (e.g. S1 Fig ewe 320B).

The gross pathology present in the placentas of the aborted ewes was associated with the presence of *C*. *abortus* organisms, as demonstrated by mZN staining of placental smears (Table 2). Similarly, the animals that lambed normally (i.e. produced viable offspring) and had focal areas of pathology on their placentas also tested positive by mZN staining of the placental smears (Table 2). Pearson’s Chi-squared permutation test revealed there was no evidence of a difference in the occurrence of any gross placental pathology between the three infected groups (P = 0.416). High numbers of organisms, as determined by qPCR, were detected in vaginal swab samples taken from ewes that aborted in groups 1–3 collected at the time of parturition and were similar to those reported in typical cases of EAE [19]. Shedding of chlamydial organisms, as determined by qPCR of vaginal swabs, was also evident in groups 1–3 in cases where clinically healthy lambs were produced, although at a reduced level compared to those animals that aborted, with a few exceptions where high numbers consistent with those associated with classical EAE were observed for strains S26/3 (ewes 320B and 210B in Table 2) and POS (ewes 2079E and 744A in Table 2). In addition, the mean number of chlamydial organisms present in vaginal swabs from ewes challenged with strain LLG (4.45x10^2^ ± 2.34x10^2^ *C*. *abortus* genomes detected per 1μl extracted total vaginal swab DNA) and which produced viable lambs was much lower than for strains S26/3 (7.48x10^5^ ± 4.49x10^5^) and POS (7.67x10^5^ ± 6.27x10^5^), although only one of the five ewes in this group showed evidence of infection compared to three of four with strain POS (Group 3) and two of four for strain S26/3 (Group 1) (Table 2). As would be expected, the bacterial loads in the challenge groups (1–3) were statistically significantly greater in comparison to the negative control group, which was below the 50 genomes cut-off for the qPCR (P<0.001). Furthermore, qPCR results were significantly higher for challenged sheep that had gross placental pathology than those that did not (P<0.001) and those that had abortion versus those that did not and versus those that had gross placental pathology (P = 0.026). Overall, when comparing animals that had evidence of bacterial shedding, the number of chlamydial genomes detected in the vaginal swabs of the S26/3 group was statistically significantly higher when compared to the bacterial load in the vaginal swabs of the LLG and POS groups (P = 0.001). Moreover, comparison of qPCR results for animals with evidence of bacterial shedding between groups 2 (LLG) and 3 (POS) showed there was a statistically significant difference at the 5% level (P = 0.026), with a greater number of genomes detected for group 3 samples.

### Histopathology

**Fig 1:**
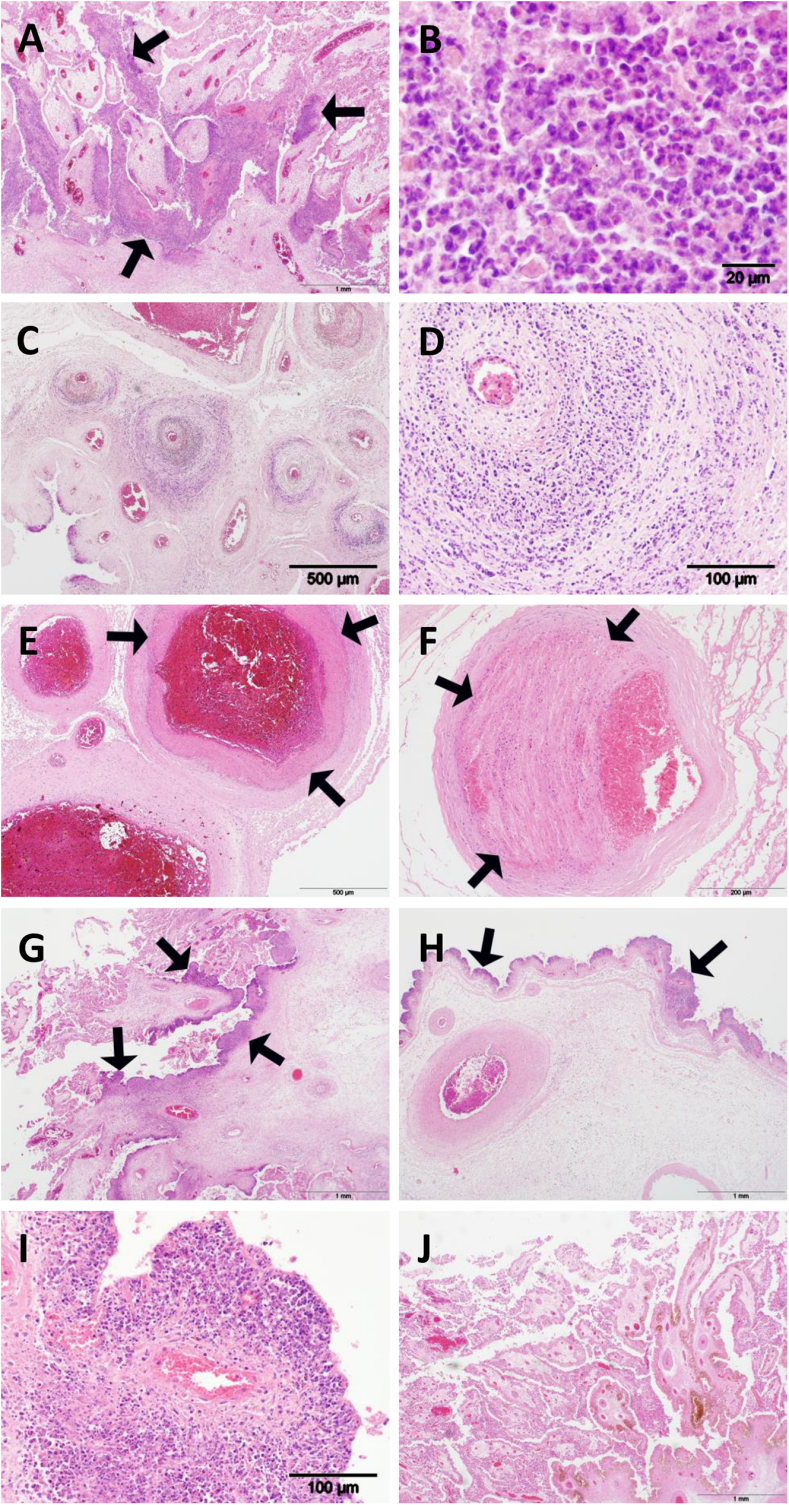
Histopathological changes in the placentas of sheep infected with *C*. *abortus* strains. (A) S26/3, note large areas of infiltration by large numbers of leucocytes into the epithelium of the cotyledon forming large amounts of necro-suppurative material (arrows); (B) S26/3, the leucocytes were mixed, primarily neutrophilic, many degenerate, inflammatory cells; (C) S26/3, arteritis as denoted by infiltration of medium to large numbers of leucocytes into the blood vessel wall, which were (D) neutrophils and fewer monocytes; (E) S26/3, severe fibrinoid necrosis as denoted by a dense band of intensely eosinophilic material within the arterial wall (arrows); (F) S26/3, early thrombosis of blood vessels. Note large thrombus comprised of palisading layers of degenerate erythrocytes, leukocytes and proteinacious material along with loss of definition of the tunica intima (arrows). (G) LLG, note less extensive infiltration of leucocytes (arrows) with less penetration into the deeper tissue compared to S26/3. (H) POS, note less extensive infiltration of leucocytes (arrows), especially the deeper tissues beneath the epithelium, compared to both S23/6 and LLG, the lesions were more associated with the intercotyledonary membrane than cotyledonary villi but of a similar morphology being comprised of mixed leucocytes but primarily neutrophils (I). (J) Negative control, note absence of inflammatory cells and necrotic material.

The placentas from S26/3-infected ewes had the most severe lesions of the three experimentally-infected groups. Histological lesions consisted of necro-suppurative placentitis as denoted by extensive destruction of the placental chorionic epithelium (trophoblast layer) which frequently had sloughed or was in the process of doing so (Fig 1A). Affected areas had been infiltrated by large numbers of densely packed mixed, primarily neutrophilic, inflammatory cells in the cotyledons (Fig 1B). A dense band of leucocytes of a similar composition was present immediately beneath the epithelium in the intercotyledonary areas and this area was often affected by arteritis (Fig 1C and 1D) sometimes resulting in fibrinoid necrosis (Fig 1E) and occasionally thrombosis formation resulting in variable degrees of occlusion of the vessel lumina (Fig 1F). The intercotyledonary area was also variably affected by oedema, haemorrhagic foci and a diffuse infiltration of mixed inflammatory cells including neutrophils and cells morphologically resembling macrophages and lymphocytes. Histological lesions in the placentas of LLG-infected animals were morphologically similar to those associated with classical EAE infection [8,19,22], however the severity was mild to moderate and the lesions rarely penetrated into the deeper tissue (Fig 1G). Additionally, vasculitis was rarely present. Placentas from Group 3 (POS) ewes also displayed morphologically similar lesions, however the lesions were less extensive than both S26/3 and LLG being more restricted to the chorionic membrane and more focal in distribution (Fig 1H) but still comprised of intense leucocyte infiltration, primarily neutrophils (Fig 1I). Vasculitis was rarely present. No significant lesions were present in the placentas from negative control ewes or in the fetal visceral tissues from any group (Fig 1J).

**Fig 2:**
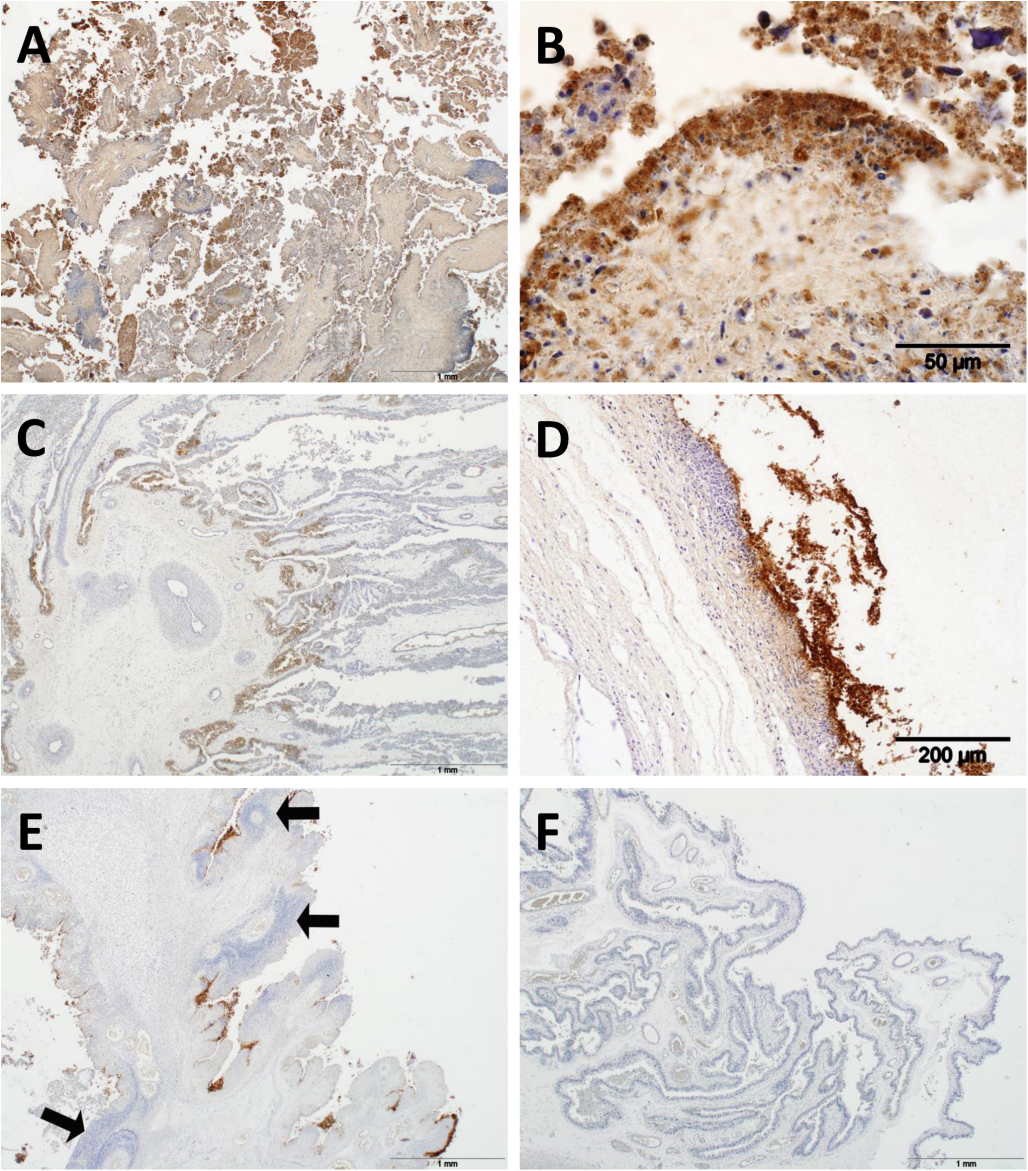
Immunohistochemical detection of chlamydial antigen in placentas. Sheep placentas infected with C. abortus strains S26/3 (A and B), LLG (C and D), POS (E) and negative control (F) and **c**ounterstained with haematoxylin. (A) S26/3, note extensive positive labelling and (B) that this was most intense and abundant in the trophoblast layer, both still attached and sloughed, and the immediately surrounding cells. (C) LLG, note positive labeling restricted to the epithelium (trophoblast) layer of the cotyledon, primarily at the bases of the cotyledonary villi and (D) in occasional foci in the intercotyledonary membrane. (E) POS, note positive labeling restricted to the epithelium (trophoblast) layer and immediately adjacent cells of the placenta in a multifocal distribution. Intense inflammatory cell infiltration, primarily neutrophils, was also present but not usually associated with the immunolabelling (arrows). (F) Negative control, note total absence of any immunolabelling and no significant infiltration by inflammatory cells.

Immunohistochemistry revealed strong positive labelling for *C*. *abortus* antigen within the cytoplasm of inflammatory cells on the surface of the placental epithelium and in trophoblast cells. Much more extensive labelling of antigen was observed in the placentas of animals that aborted in the S26/3 group compared to those in the other two challenged groups (Fig 2A). Strong antigen labelling was observed in the epithelium and, while much of the trophoblast layer was denuded, the remaining trophoblast cells, either still attached to the cotyledon or in the process of sloughing, labelled intensely for *C*. *abortus* antigen (Fig 2B). Placentas from the LLG infected animals (Group 2) mostly showed antigen labelling restricted also to the placental trophoblast/ epithelial layer of the cotyledons especially at the base of the cotyledonary villi (Fig 2C) but when foci of labelling were present in the intercotyledonary membrane the labelling was intense (Fig 2D). Group 3 (POS) animals showed a more multi-focal distribution of antigen labelling, again this was primarily restricted to the trophoblast layer and sloughed cells (Fig 2E). No labelling of *C*. *abortus* antigen was observed in any of the negative control ewes (Fig 2F). Additionally, no labelling was observed with any of the negative control preparations from the placental tissues of the challenge groups, while all positive control preparations showed strong, positive labelling.

### Serological analysis

**Fig 3:**
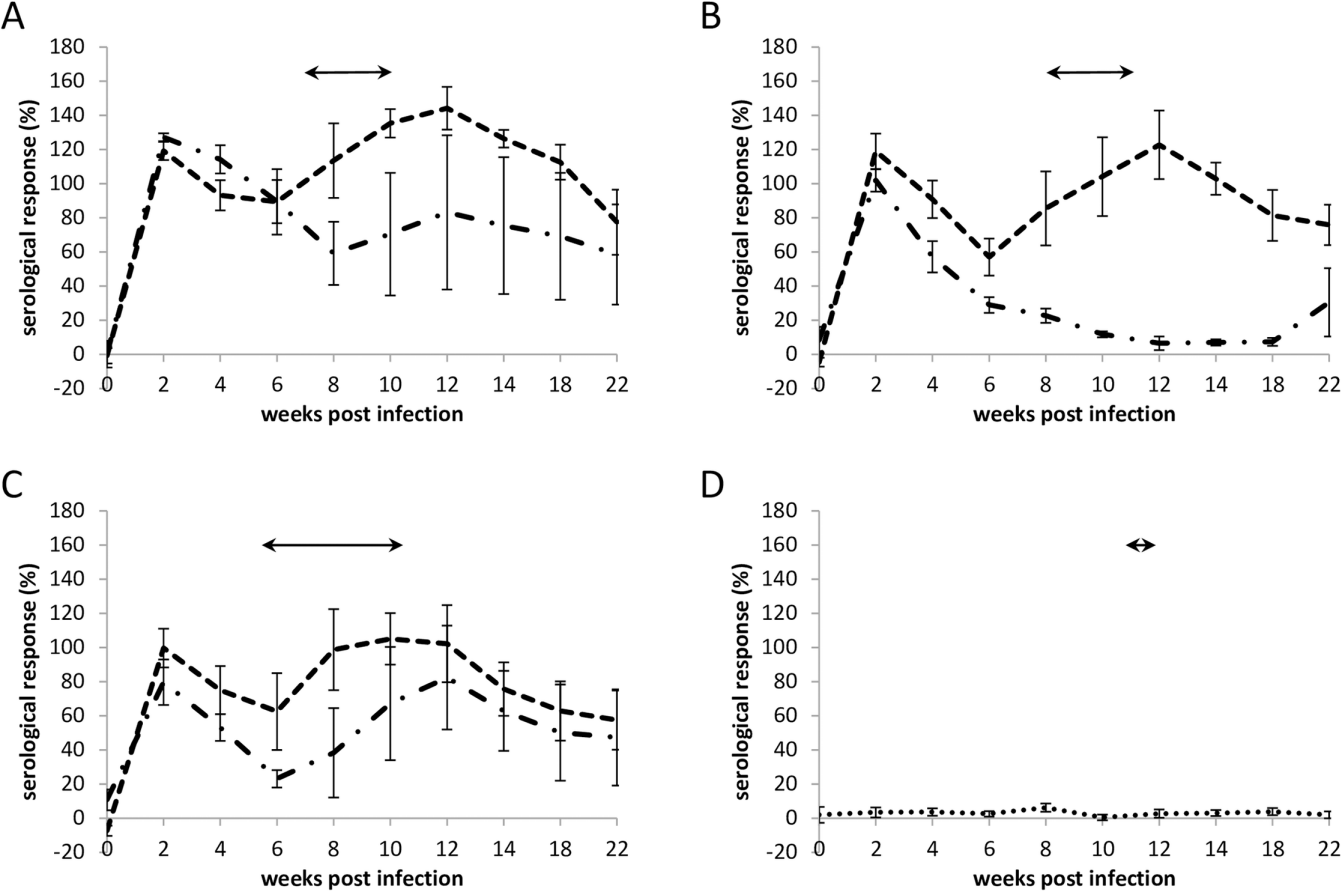
Serological responses to *C*. *abortus* strains. Detection of *C*. *abortus* antibody in ewes that aborted (includes non-viable births) (-—-) and lambed (-. -) following challenge with *C*. *abortus* strains S26/3 (A), LLG (B) and POS (C) or with negative control inoculum (D). Means (± SEM) of normalised responses (see Materials and Methods) are shown. 100% is equivalent to an OD450nm of 2.25. The lambing/abortion period is indicated by the horizontal double-headed arrows.

The mean antibody responses detected for the aborted and lambed animals in each of the four groups are depicted in Fig 3. All ewes were confirmed seronegative prior to challenge (day 0). Following challenge, similar antibody levels were observed in the aborted animals in all 3 infection groups. Initially, a rapid increase in antibody titre was observed two weeks post-challenge followed by a gradual decline until around six weeks post-challenge. Thereafter, the antibody response was found to increase, peaking at 10–12 weeks post-challenge, which coincided with abortion. A statistically significant difference in mean antibody response was found between ewes which aborted versus ewes which lambed normally (P<0.001) and between all animals that exhibited gross placental pathology versus those that showed no signs (P<0.001). When considering all animals that exhibited gross placental pathology, no statistically significant difference in the mean antibody responses was observed compared with just the animals that aborted (P = 0.256). In ewes which lambed normally the antibody trends were similar in Groups 1 (S26/3; Fig 3A) and 3 (POS; Fig 3C), mirroring those of the animals that aborted but with a lower level of response, reflecting the reduced pathology and lower bacterial load observed in these animals. Group 2 (LLG) lambed animals displayed a different antibody profile and showed that after an initial response to challenge (Fig 3B), the titre gradually decreases over time to levels observed prior to challenge, which reflects these animals being mostly negative or having only low levels of chlamydial antigen present in the placentas. As expected, the negative control group (Fig 3D) had a significantly lower mean antibody response (background level) compared to challenged groups (S26/3, LLG and POS) [21] (P<0.001). Overall, ewes infected with S26/3 had a significantly higher mean antibody response versus LLG and POS (P<0.001). However, there was no significant difference between LLG and POS (P = 0.112) and there was no evidence of a difference in the mean antibody response of ewes that aborted versus ewes that lambed normally across comparison groups. Indeed, none of the interactions investigated between these groups were significant.

**Fig 4:**
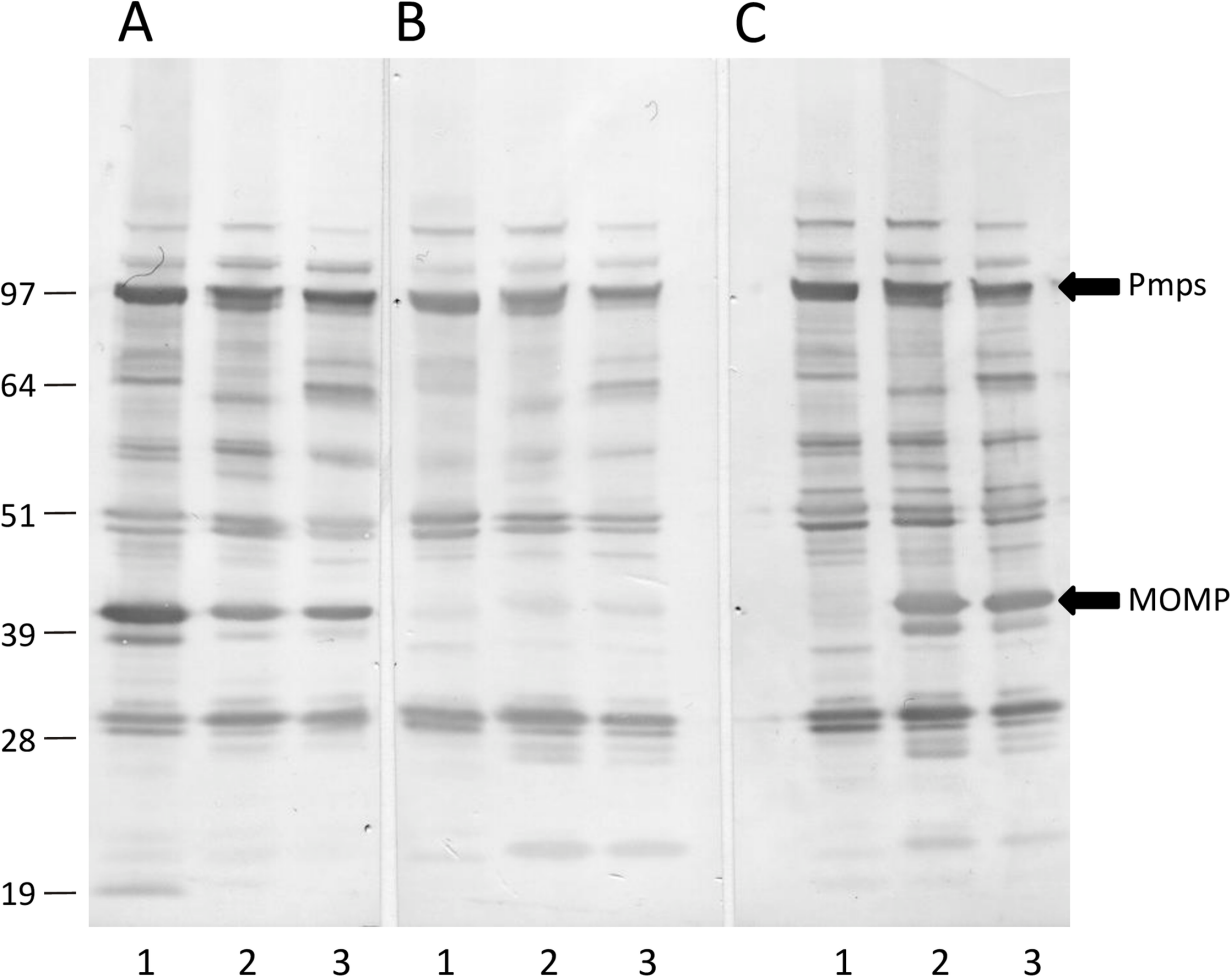
Immunoblot analysis of *C*. *abortus* EB preparations. EBs prepared from *C*. *abortus* strains S26/3 (lane 1), LLG (lane 2) and POS (lane 3) were run on 4–12% x NuPAGE Bis-Tris polyacrylamide gels under reducing conditions and immunoblotted with pooled post-abortion sheep serum from ewes infected with *C*. *abortus* strains S26/3 (A), LLG (B) and POS (C) as detailed in Materials and Methods. Molecular masses (kDa) are as indicated.

Immunoblot analysis of S26/3, LLG and POS EBs with pooled convalescent sera from the ewes that aborted in the three experimental groups revealed differences in seropositivity, specifically to MOMP (Fig 4). S26/3 MOMP was recognized strongly by sera from S26/3-infected animals, while weaker anti-MOMP reactivity was observed when the sera were used to probe LLG- and POS EBs. Interestingly, there was no apparent reactivity to LLG MOMP with sera from LLG-infected animals, i.e. there was no self-recognition of linear epitopes. Additionally, sera from LLG-infected animals also did not recognize MOMP in either POS or S26/3 EBs. On the other hand, only POS and LLG MOMP were recognized by sera from POS-infected animals (although weakly), with no reactivity to S26/3 MOMP being apparent. Similar results were observed for sera from the ewes that lambed, although somewhat reduced in positivity in comparison. Additionally, there was no observed reactivity to MOMP for any of the strains with sera from POS-infected animals that lambed (results not shown). No positivity was observed to EBs from any of the strains with sera from the negative control group (results not shown).

Additional dot blot analysis was carried out with S26/3, LLG and POS EBs, each spotted onto nitrocellulose membrane and probed with convalescent sera from the three experimental groups. Strong reactivity of similar intensity was observed with all three *C*. *abortus* strains when probed with pooled convalescent sera from each of the three groups. Dot blot analysis was repeated with COMCs prepared from EBs from each of the three strains and although the strength of signal was slightly reduced, no differences were observed in the reactivity between antigens or the sera from the three groups (results not shown).

## Discussion

In this study, we describe the pathogenesis and clinical outcome of an experimental infection in pregnant sheep of the two variant strains of *C*. *abortus*, LLG and POS. Until now, *in vivo* studies on these variant strains have only been conducted in mice [10,15,29], and although differences have been observed with regards to virulence and pathogenicity, mouse placentation differs greatly from that of the sheep and it was therefore unknown how these strains would behave in the natural small ruminant host. Following experimental challenge, we found that infection of pregnant ewes with these two variant strains and the wild-type UK reference strain S26/3 resulted in comparable abortion rates and gestational lengths. Gross placental pathology within each of the groups and across the three groups was extremely variable, as observed previously [1], with little to note in terms of distinguishing the variant strains LLG and POS from the classical wild-type strain S26/3, with an exception being two aborting ewes challenged with stain LLG showing limited areas of pathology. This suggests that clinically, at least in terms of pregnancy outcome and gross placental pathology, the infections resulting from these two variant strains are very similar to those resulting from typical wild-type strains. However, we did note that in the placentas from ewes that were challenged with strain LLG and produced viable lambs, only one of the five showed any evidence of gross pathology or organisms in placental smears, and even then the bacterial shedding in the ewes in this group was considerably lower overall than in similar lambed animals from the other two groups, suggesting that infection with strain LLG is milder in nature. We also noted a perceived increase in the retention times of the placentas for the ewes infected with LLG and POS compared to S26/3, a phenomenon reported commonly in goats rather than sheep [30], and which probably also accounts for the more necrotic state of some of the expelled fetuses and placentas observed with these two strains.

Despite the similarity in clinical outcome, prominent differences were observed in the severity of placental pathology for the three strains. Compared to strain S26/3, which exhibited the most severe histological lesions of the three strains and the most extensive labelling of chlamydial antigen, infection was considerably milder for strain LLG, while infection for strain POS was the least extensive and mostly restricted to small foci. For strain LLG, this reduction in gross pathology and the reduced organism load evident in placental smears, was correlated to a significant reduction in organisms shed at parturition. Similar observations with regard to severity of infection and colonization of the placenta have been noted in mice [31]. Indeed, strains LLG and POS have been shown to cause abortion and reduced litter size in mice despite reduced colonization of the placenta when compared to other *C*. *abortus* strains [15]. This suggests that the severity of placental pathology, the number of organisms present and the destruction of placental cells alone do not necessarily correlate with an outcome of abortion in pregnant sheep. Thus, other factors, such as the degree of placental arteritis, involvement of pro-inflammatory cytokines and differences in antibody reactivity may play an important role in pregnancy outcome for these strains.

Following investigation of the serological responses of the infected ewes, we found prominent differences in reactivity, in particular to MOMP, in the three *C*. *abortus* strains when probed with convalescent sera from the challenged ewes. In this study, we observed no seropositivity with the anti-LLG sera against the MOMP of any of the three strains, which contrasted with the strong reactivity of the anti-S26/3 sera with S26/3 MOMP and weaker positivity with both LLG and POS MOMP. These results confirm previous observations that variant *C*. *abortus* strains differ in their seroreactivity to MOMP [9]. Furthermore, we observed that the seroreactivity to POS MOMP was different to that of LLG MOMP, showing some seropositivity with the anti-POS sera against POS and LLG MOMP. In particular, we noted the lack of ‘self recognition’ with LLG MOMP when probed with anti-LLG sera following denaturing polyacrylamide gel electrophoresis. However, repeating the analyses by dot blot using whole EBs and COMCs, which comprises mostly MOMP and in which the MOMP retains its native conformational trimeric structure [26,27], resulted in identical results for each of the pooled sera against each of the antigens from the three strains. Taken together these results highlight the pronounced conformational nature of the MOMP epitopes in the variant strains LLG and POS, compared to S26/3, which are susceptible to heat denaturation. Important differences observed in the amino acid sequences of the MOMP of the wild-type strain S26/3 and the variant strain LLG might have resulted in mostly conformational epitopes only being present in the MOMP of the variant strain. Indeed, differences in the surface exposed amino acids of the oligomeric MOMP sequence of the LLG strain in variable segments (VSs) 1, 2 and 4 compared to strain 577 (identical to S26/3) have previously been shown to ablate the binding of two neutralizing and conformationally-dependent monoclonal antibodies that specifically recognize strain 577 [32].

While differences in MOMP conformational epitopes may explain the differences between the three strains, recent whole genome sequence analysis of the two variant strains has shown the MOMP from strains LLG and POS to be identical in primary amino acid sequence (Unpublished work) (S4 Fig). Additionally, these two variant strains, which are described as homologous strains in the literature, have been shown to possess similar monoclonal reactivity and growth characteristics [9], although this and other studies indicate that there may be differences [9,15]. The reasons for these apparent discrepancies are unclear. The fact that these strains have arisen in different locations in the highland areas of Macedonia in Greece in different animal species, and are still circulating two decades later in a lowland region of Macedonia [17], supports the possibility that they may be distinct. However, the recently completed whole genome sequence analysis shows that LLG and POS are identical in at least 99% of the genome (Unpublished work) with the caveat that minor variation could be present in two regions that have not been closed. However, these two regions encompass four of the polymorphic membrane proteins [33], and any slight variation in these highly polymorphic genes could result in changes in surface expression which could impact on the antibody recognition of the MOMP through possible steric hindrance, which is one alternative explanation for the serological results we observed in this study.

The differences and lack of apparent seroconversion to MOMP for the variant strains has implications for the diagnosis of *C*. *abortus* infection, particularly when relying on denatured MOMP-based tests, which could result in false-negative results. Similarly, such differences in MOMP and/or other chlamydial antigens could have important implications on the design of any new-generation recombinant subunit *C*. *abortus* vaccines, impacting on protective efficacy. Indeed, it has already been shown that vaccination with the live-attenuated 1B *C*. *abortus* vaccine strain is less effective at reducing placental and fetal colonization in mice infected with LLG and POS in contrast to another virulent *C*. *abortus* strain [29], implying differences in terms of cross-protection.

## Conclusion

This is the first study to investigate the pathogenesis of two known variant strains of *C*. *abortus* in small ruminants, the natural host animal. Comparison with a typical wild-type strain, has revealed that although they behave similarly in terms of clinical outcome there are some important differences observed in gross and histological placental pathology, colonization and antibody reactivity to MOMP. The results clearly suggest that gross placental pathology, while generally correlating with abortion in pregnant ewes, is not indicative of colonization and the severity of infection. Additionally, they suggest that there are important conformational differences in MOMP epitopes that could impact on diagnostic test and vaccine efficacy. Whole genome sequencing and comparative analysis of LLG and POS with typical wild-type *C*. *abortus* strains may reveal gene differences that are responsible for the differences between the strains and provide new targets for controlling these infections.

## Supporting information

S1 Fig Photographs of placentas from ewes challenged with *C*. *abortus* strain S26/3. Length of gestation, mZN status and bacterial load expressed as IFU per 1 μl extracted placental material are shown on each photograph.
(TIF)

S2 Fig Photographs of placentas and fetuses from ewes challenged with *C*. *abortus* strain LLG. Length of gestation, mZN status and bacterial load expressed as IFU per 1 μl extracted placental material are shown on each photograph.
(TIF)

S3 Fig Photographs of placentas and fetuses from ewes challenged with *C*. *abortus* strain POS. Length of gestation, mZN status and bacterial load expressed as IFU per 1 μl extracted placental material are shown on each photograph.
(TIF)

S4 Fig Amino acid sequence alignment of the major outer membrane protein of *C*. *abortus* strains S26/3, LLG and POS. Amino acid changes in MOMP at positions 96 (Asn to Asp), 165 (Ile to Val), 286 (Ser to Gly), 326 (Ala to Thr) and 330 (Ser to Asn) are shown.
(TIF)
