# Supplementary figures and images for: Pathogenic outcome following experimental infection of sheep with Chlamydia abortus variant strains LLG and POS
Source: PLoS One. 2017 May 11;12(5):e0177653. doi: 10.1371/journal.pone.0177653 (PMC5426687; doi:10.1371/journal.pone.0177653)

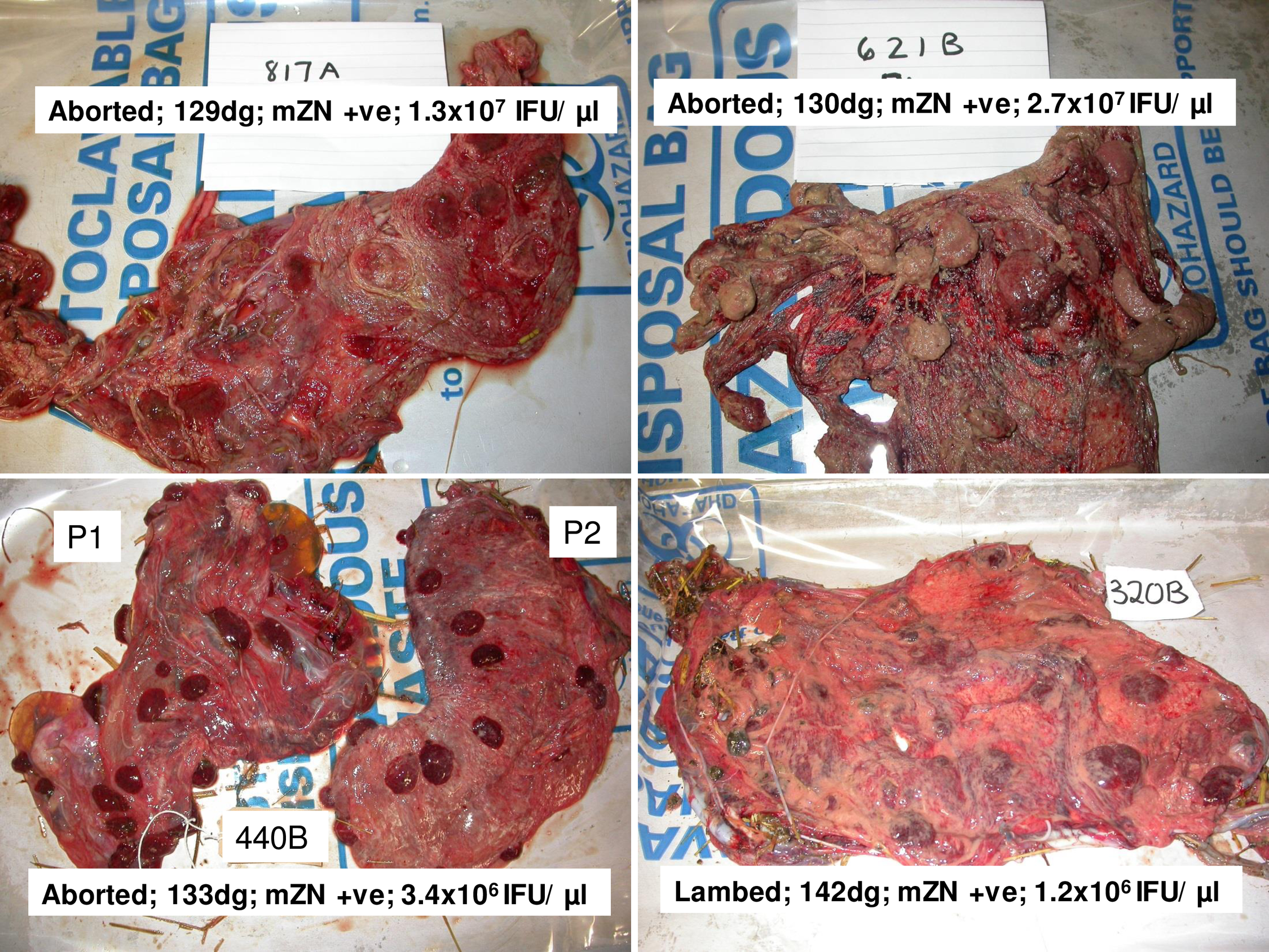

Supplement: S1 Fig — Length of gestation, mZN status and bacterial load expressed as IFU per 1 μl extracted placental material are shown on each photograph. (TIF) [file pone.0177653.s001.tif]

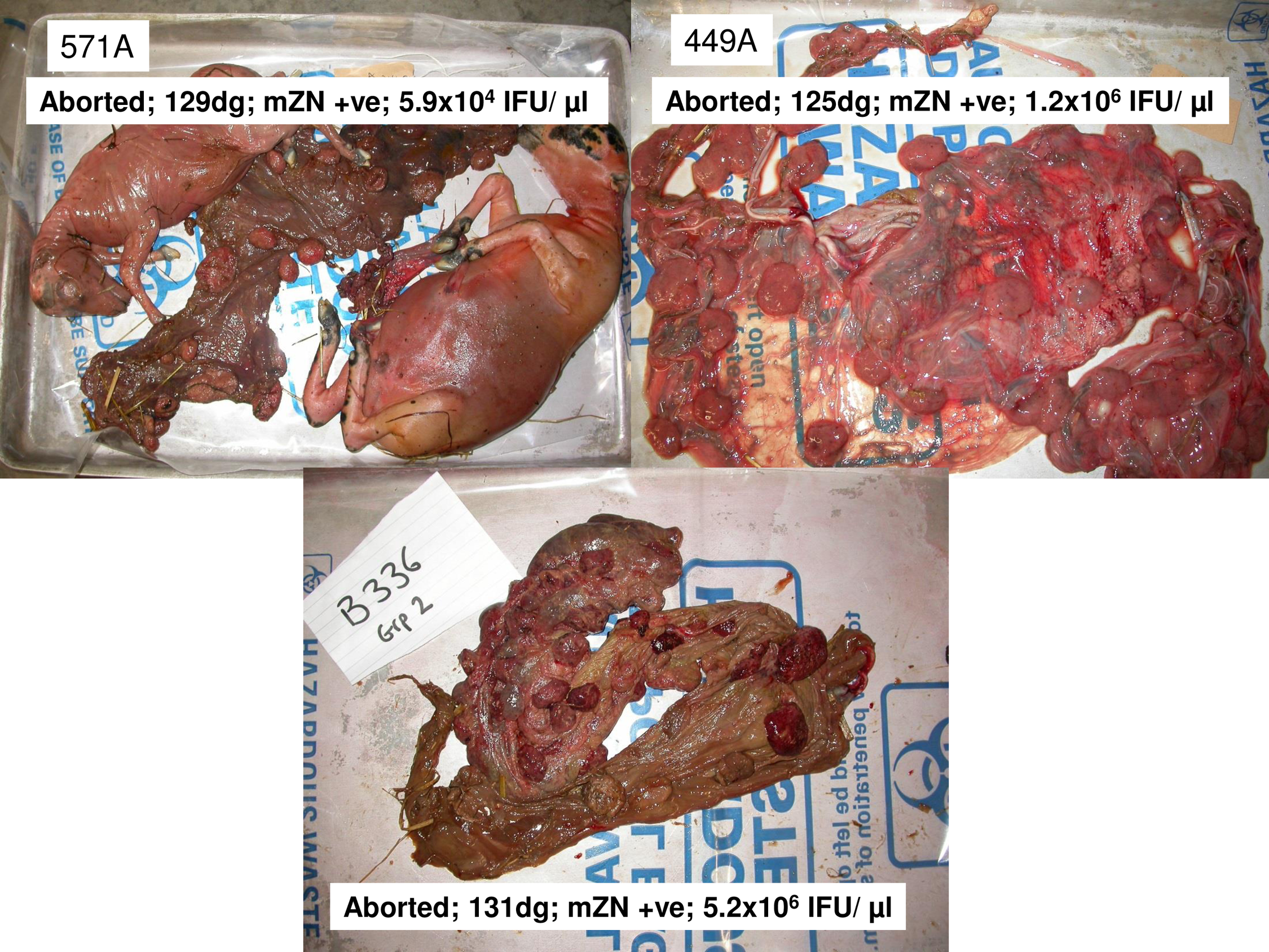

Supplement: S2 Fig — Length of gestation, mZN status and bacterial load expressed as IFU per 1 μl extracted placental material are shown on each photograph. (TIF) [file pone.0177653.s002.tif]

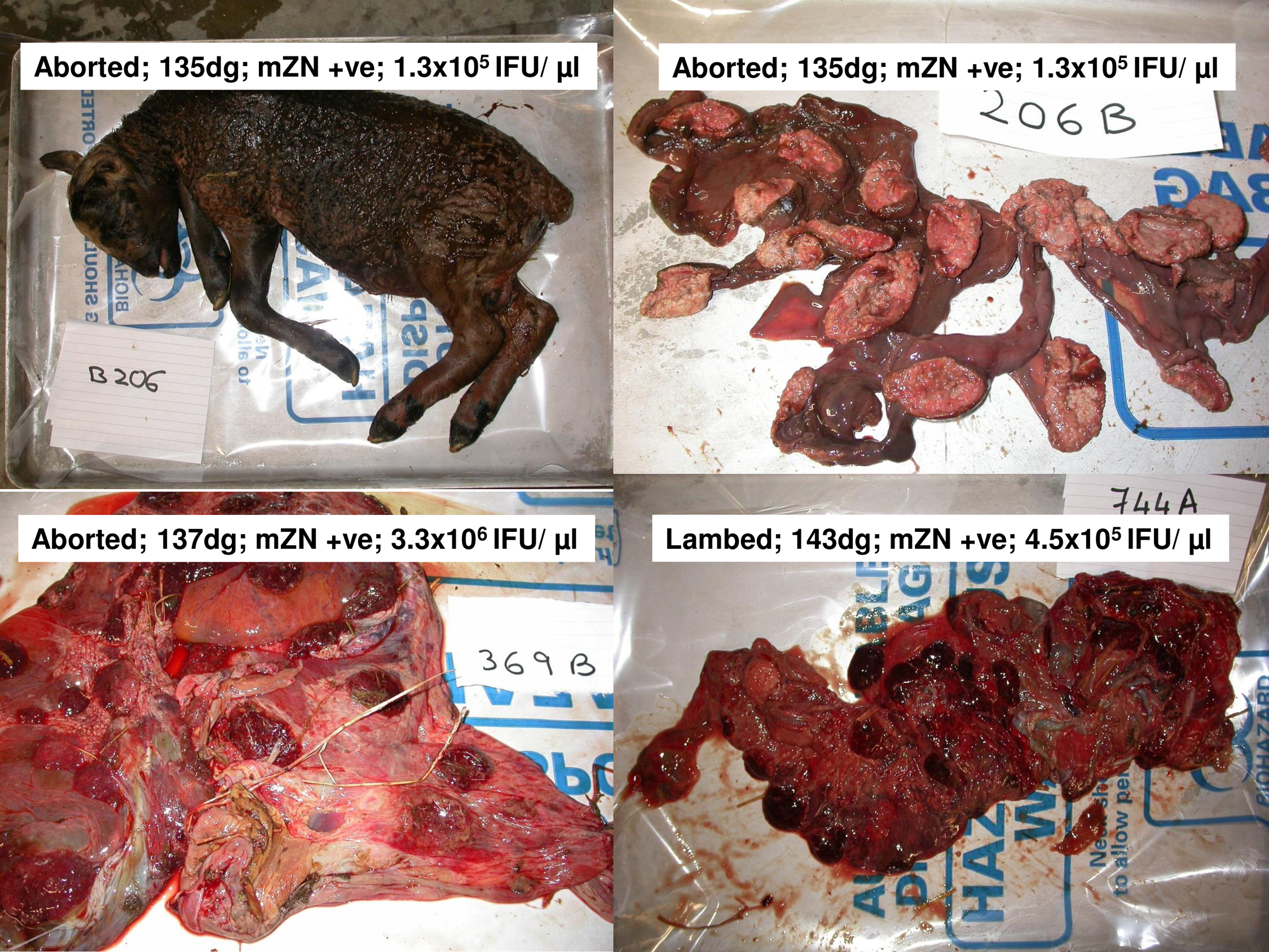

Supplement: S3 Fig — Length of gestation, mZN status and bacterial load expressed as IFU per 1 μl extracted placental material are shown on each photograph. (TIF) [file pone.0177653.s003.tif]

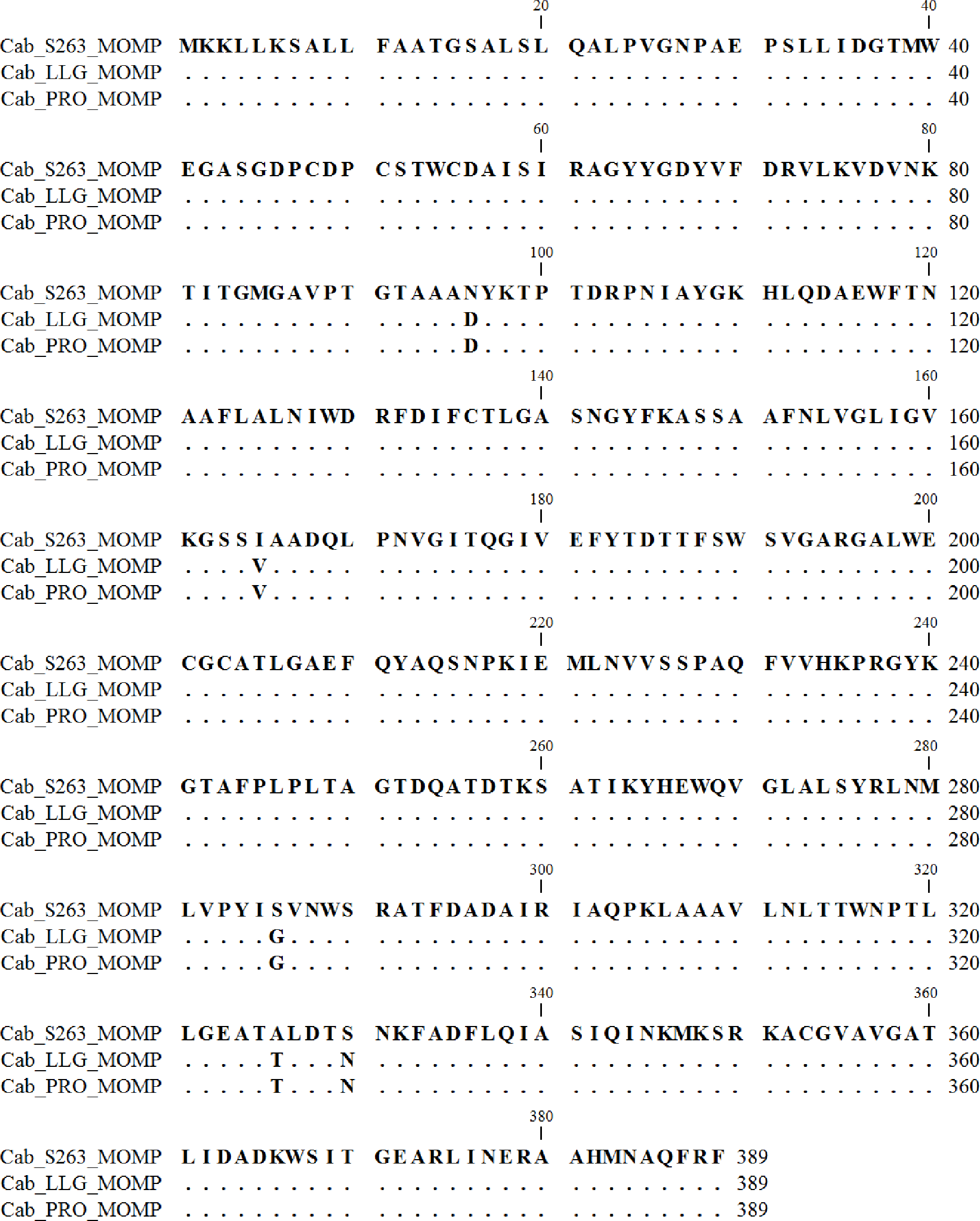

Supplement: S4 Fig — Amino acid changes in MOMP at positions 96 (Asn to Asp), 165 (Ile to Val), 286 (Ser to Gly), 326 (Ala to Thr) and 330 (Ser to Asn) are shown. (TIF) [file pone.0177653.s004.tif]
